# Fertility trends and selective fertility in Wales during the COVID-19 pandemic: a population-level time series analysis

**DOI:** 10.1136/bmjph-2025-004632

**Published:** 2026-11-06

**Authors:** Lucy Stone, James Rafferty, Ian Farr, Catherine A Thornton, Lucy J Griffiths

**Affiliations:** 1 Population Data Science, Swansea University Medical School, Swansea, UK; 2 Biomedical Sciences, Swansea University Medical School, Swansea, UK

**Keywords:** COVID-19, Demography, Sociodemographic Factors, Maternal health

## Abstract

**Introduction:**

Measures put in place during the COVID-19 pandemic caused substantial social and economic disruptions, influencing fertility intentions and fertility patterns. Yet not much is known about how the pandemic impacted fertility in the UK by socioeconomic background.

**Methods:**

This population-based study used 220 025 live births born between April 2015 and April 2023 aggregated into 4-week periods from the Secure Anonymised Information Linkage databank to calculate general fertility rates (GFRs) for Wales. Interrupted time series and counterfactual analyses were conducted using Autoregressive Integrated Moving Average models that were also stratified by maternal age and Welsh Index of Multiple Deprivation (WIMD).

**Results:**

GFRs drifted from their original time series with a significant abrupt but temporary decline of 0.22 (95% CI −0.35 to −0.10) per 4-week period between December 2020 and April 2021. Notably, those aged 15–19 years old had an abrupt, permanent decline in GFRs of 0.16 (95% CIs −0.29 to −0.03) per 4-week period up to April 2023 with no upturns in GFRs throughout the whole time series. Those living in the most deprived quintile had an abrupt, temporary decline in GFRs of 0.20 (95% CI −0.36 to −0.04) per 4-week period between December 2020 and August 2021, a longer duration than any other WIMD quintile. There was no significant change in the time series for those living in the least deprived quintile, nor for those aged 30–34 years old.

**Conclusions:**

The study found that the COVID-19 pandemic adversely affected fertility more for younger age groups and those living in more deprived areas. As these groups have higher risks of adverse maternal and neonatal outcomes, selective fertility might have produced a healthier cohort of mothers and babies less likely to experience poor maternal and neonatal health outcomes.

**WHAT IS ALREADY KNOWN ON THIS TOPIC**

- Many countries experienced a drop in fertility rates 9 months after the start of the COVID-19 pandemic, followed by upturns in rates before levelling off to pre-pandemic levels.
- Selective fertility by socioeconomic and demographic characteristics has been found in some European countries, but little evidence exists in the UK.

**WHAT THIS STUDY ADDS**

- This study found that the COVID-19 pandemic adversely impacted fertility for people in numerous age groups and deprivation quintiles, namely younger people (aged 15–19 years old) and those living in the more deprived areas, whereas fertility for those aged 30–34 and those living in the least deprived areas was largely unchanged.

**HOW THIS STUDY MIGHT AFFECT RESEARCH, PRACTICE OR POLICY**

- As young people and those from less advantaged backgrounds have higher risks of adverse maternal and neonatal outcomes, selective fertility might have produced a healthier cohort of mothers and babies less likely to experience poor maternal and neonatal health outcomes during the pandemic.

## Introduction

 Changes to fertility patterns, namely the number of live births, are commonly observed in crisis situations. Events that cause significant social instability including epidemics and pandemics have marked effects on the number of births 9 months later.^1,2^ Explanations for a decline in live births during times of crisis are often behavioural, reflecting alterations to fertility intentions.^3,4^ To limit transmission of SARS-CoV-2, the UK and Welsh Governments implemented both national and regional restrictions, including lockdowns, the closing of public spaces, school closures and alterations to health service provision.^5^ Intended to protect population health, these measures caused substantial social and economic disruptions and uncertainty significantly influencing fertility intentions of those of reproductive age, with many choosing to postpone or halt childbearing, especially initially.^6–9^

Early assessments of live births 9 months after the start of the COVID-19 pandemic in March 2020 revealed a decline in birth rates in some countries including France and the USA, though only significant in a few countries, notably Hungary, Italy and Spain.^10^ In Europe, drops in fertility rates of between 0.5% and 11.4% have been found, alongside a 14% drop in live births 9–10 months after the start of the pandemic.^11,12^ In the USA, a decline in average monthly fertility rates was found 9 months following wave 1 of the pandemic, with a reversal following wave 2, although these patterns varied by state.^13^ Studies using longer term data show similar trends of reductions in fertility rates 9 months after the start of the pandemic followed by significant upturns from March 2021 and a continued upturn throughout 2021 for England and Wales, other Western European countries and the USA.^14^ These temporary reductions and subsequent revivals in fertility might be explained by changes to fertility intentions at the start of the pandemic and the postponement of conceptions to later in 2020 or 2021. More recent studies found further unexpected drops in fertility in 2022. This has been linked to the easing of restrictions and the rollout of national vaccination programmes; increasing mobility and the return to normality have contributed to declining birth rates in some European countries alongside initial uncertainty surrounding the COVID-19 vaccination and pregnancy.^15,16^

Fertility intentions and decision-making are often a complex process influenced by several socioeconomic, political and individual factors ranging from income, education level, gender equality, living situations and levels of support.^17,18^ Increased economic and social uncertainty during the COVID-19 pandemic was expected to affect fertility differently for different populations resulting in depressed fertility in high-income societies especially.^10,19^ This paired with the exacerbation of pre-existing inequalities and the unequal effect of mitigation measures on individuals’ socioeconomic situations could disproportionately influence fertility intentions for those with specific socioeconomic and demographic backgrounds.^20,21^ Women with differing socioeconomic and demographic backgrounds were likely to experience varying risks of exposures and heterogeneous fertility responses during the pandemic.^3,9,21^ Selective fertility has been found in Spain with reduced fertility rates for younger people.^22^ Likewise, Nordic countries consequently experienced higher fertility rates 9 months after the start of the pandemic that were largely driven by women aged 28–35 and women who already had children.^23,24^

Research to assess selective fertility during the pandemic in the UK remains scarce. One descriptive study using live births data from Scotland up to 2021 found fewer live births 9 months after the start of the pandemic among women living in the most deprived areas and an increase in live births among those in the least deprived.^25^ To better understand how the pandemic affected fertility in the UK, we have used linked population-scale data from around 220 000 singleton live births between April 2015 and April 2023. This study has assessed changes to fertility patterns during the pandemic in Wales, focusing on effects of maternal age and deprivation status to examine if trends differ for subgroups.

## Materials and methods

### Data sources and study cohort

This study used anonymised individual-level population-based data accessed via the Secure Anonymised Information Linkage (SAIL) Databank, the national Trusted Research Environment for Wales.^26–28^ The SAIL Databank contains extensive anonymised health and administrative data about the population of Wales, accessible in anonymised form via a secure data sharing platform, all underpinned by an innovative and proportionate Information Governance model.^26–28^

The following datasets were linked to develop the study cohort: the Annual District Birth Extract (ADBE) from the Office for National Statistics (ONS)—a register of all births collected from birth registrations,^29^ the Maternity Indicators Dataset (MIDS) that collects maternal data at initial antenatal assessment and maternal and neonatal data at labour and birth,^30^ and the Welsh Demographic Service Dataset (WDSD) that gathers demographic characteristics of all people interacting with any NHS Wales healthcare service, providing demographic details including measures of deprivation.^31^ During the anonymisation process of data sources within the SAIL Databank, individuals are assigned an anonymised linking field (ALF) based on their NHS number, name, sex, date of birth and postcode.^27^ ALFs were used to link the datasets outlined above. We did not have access to personal identifiable data.

The study cohort started with live births from the ADBE which were then linked to MIDS. The data were filtered to only include births where mothers have an initial assessment record and singleton birth and were then linked to the WDSD. Finally, the data were filtered to include mothers who were of reproductive age at birth (15–49 years) and live births with a week of birth date between 1 April 2015 and 18 April 2023. The week of birth date is the date of the Monday that occurred before or on the actual date of birth. The final time series included live births born to women of reproductive age registered as residents in Wales (figure 1).

**Figure 1:**
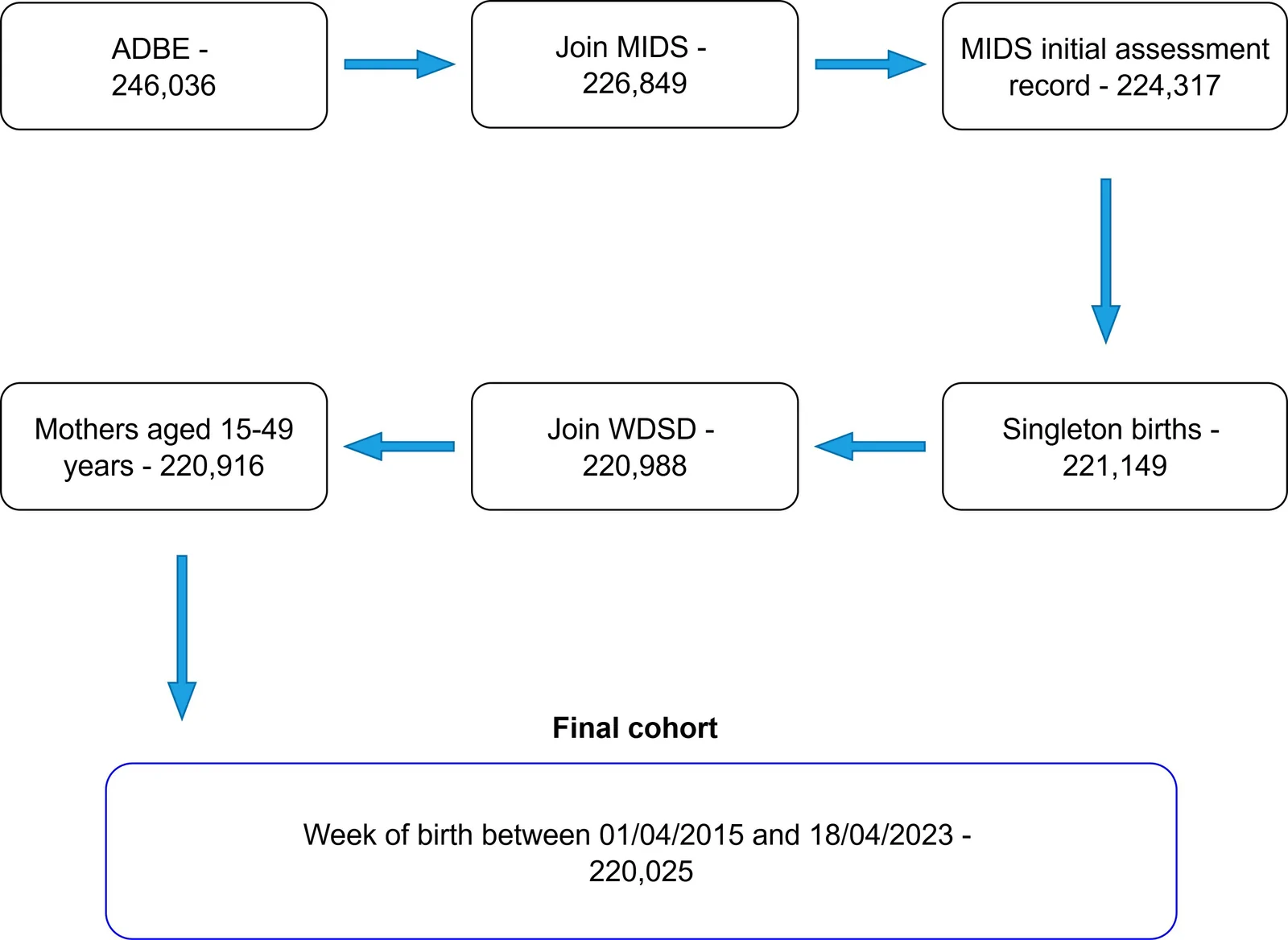
Consort diagram and final cohort. ADBE, Annual District Birth Extract; MIDS, Maternity Indicators Dataset; WDSD, Welsh Demographic Service Dataset.

### Patient and public involvement

No patients or members of the public were involved in this study.

### Analytical approach

Live births were aggregated into 4-week periods starting from the period between 1 and 28 April 2015 and ending on the period 22 March to 18 April 2023. General fertility rates (GFRs) were calculated for each 4-week period using the number of live births divided by the number of women of reproductive age estimated by linear interpolation of mid-year population data of the female population between 2014 and 2023 from the WDSD. These were then multiplied by 1000 giving GFR per 1000 women.

Analysis was also stratified by age of mother at birth of the baby and the WIMD 2019 quintiles. Lower-layer super output areas (LSOAs) are small geographic units in Wales, each comprising approximately 1600 individuals. WIMD assigns an area-level deprivation score to every one of the 1909 LSOAs in Wales and ranks them from most—1 to least—1909 deprived.^32^ These scores are then divided into five quintiles of deprivation levels for research purposes. Age categories were 15–19, 20–24, 25–29, 30–34, 35–39 and 40–49 years and WIMD quintiles ranged from quintile 1 (most deprived) to quintile 5 (least deprived). Analysis was also going to be stratified by parity but due to the extent of missing data, this was not included. The COVID-19-related interruption period for fertility starts from December 2020, 9 months after the first national lockdown commenced in the UK on 23 March 2020.

Interrupted time series (ITS) analyses were conducted using seasonal Autoregressive Integrated Moving Average (ARIMA) models to test the impact of the COVID-19 pandemic on GFRs. The seasonal ARIMA model is expressed as ${\left(p,d,q\right)x(P,D,Q)}_{S=13}$ where *s*=13 refers to the seasonal period, which corresponds to 13 4-week periods present in 1 year.^33^ *d* indicates the order of non-seasonal differencing and *D* the order of seasonal differencing. As GFRs are seasonal and followed a downward trend in most cases, *d* and *D* in each model were 1. The autoregressive part of the model is represented by *p* and *P*, and *q* and *Q* represent the moving average (MA) component of the model.^33^ For example, the model ${\left(0,1,1\right)(0,1,1)}_{13}$ was chosen for the first model indicating an MA model with *q* lags=1 and *Q* lags=1. The ARIMA models that best fit the time series were identified using automated model selections and manually selected models identified using plots of the autocorrelation function (ACF) and partial autocorrelation function (PACF). The best models were selected using Akaike Information Criterion. Each model was tested for trends, autocorrelation and seasonality using ACF and PACF plots alongside residual plots and the Ljung-Box test to examine remaining autocorrelation and to determine the validity of each model.^33^ The model specification for all models is shown in online supplemental table S1.

Separate ARIMA models were run for Wales and for each age group and WIMD quintile. Once the most suitable models were identified, the interruption impact variables were added which included (1) a step variable which took the value of 0 prior to the start of the interruption period (December 2020) and 1 afterwards to measure an abrupt sustained impact and (2) a pulse variable which took the value of 0 prior to the interruption period, 1 during the specific interruption period and 0 afterwards to measure an abrupt temporary impact.^33^ To assess the length of the interruption impact, multiple pulse variables were examined. Models tested a pulse variable for December 2020 then subsequently added a 4-week period onto the pulse variable until the penultimate 4-week period starting in February 2023. Based on a hypothesis developed a priori, the COVID-19 pandemic was expected to have an abrupt temporary impact with GFRs reaching near baseline levels as the pandemic wore on. An abrupt sustained impact was also tested to ascertain if the impact on GFRs was more permanent than initially expected. The same goodness-of-fit tests as outlined above were then used to assess the appropriateness of each model with an interruption impact variable.

The same ARIMA models and tests were also used on the pre-pandemic time series to forecast the GFRs that would have occurred if the pandemic had not taken place during that time (the counterfactual analysis). Models were used to calculate the expected GFRs and 90% CIs for each 4-week time period between December 2020 and March/April 2023. The relative difference between the total observed and expected GFRs between December 2020 and March/April 2023 was also calculated.

The SAIL Databank was interrogated using Database 2 Structured Query Language. All analysis was conducted using R V.4.2.2 (R Foundation for Statistical Computing, Vienna, Austria).

## Results

The time series consisted of 220 025 live births; 159 250 were born pre-pandemic and 60 775 were born during the pandemic period. Before the pandemic, GFRs were declining in Wales, which was consistent across most of the stratified groups except for those aged 40–49 years old and those living in the least deprived areas (online supplemental figure S1). As seen in table 1, across the study period, the mean pre-pandemic GFR in Wales was 3.12 births per 1000 women of reproductive age (SD=0.15), with a modest decline during the pandemic (mean=2.89, SD=0.18). Age-specific GFRs were highest among women aged 25–29 before the pandemic (mean=6.33, SD=0.45) with the highest rates observed in the 30–34 age group during the pandemic (mean=5.98, SD=0.40). SD were generally low to moderate, indicating consistent fertility patterns within age bands, though younger age groups showed greater variability. The most deprived quintile consistently exhibited higher mean GFRs with a pre-pandemic mean of 3.79 (SD=0.26) and 3.27 (SD=0.20) during the pandemic, while the least deprived had lower GFRs (pre-pandemic mean=2.70, pandemic mean=2.60). SD were slightly higher in more deprived areas, suggesting greater heterogeneity in these groups. Pandemic-related declines were observed across all groups except for those aged 40–49. The steepest drops in mean GFRs were among those in the younger age groups (15–19, 20–24 years) and those in the most deprived quintile.

**Table 1:** Mean pre-pandemic (April 2015–November 2020) and pandemic (December 2020–April 2023) GFRs and SD for Wales and by age of mother and WIMD quintile

Group	Pre-pandemic		Pandemic	
	Mean	SD	Mean	SD
Wales	3.12	0.15	2.89	0.18
Age				
15–19	1.02	0.15	0.69	0.09
20–24	3.84	0.31	3.20	0.28
25–29	6.33	0.45	5.73	0.50
30–34	6.24	0.40	5.98	0.40
35–39	3.31	0.23	3.12	0.33
40-49	0.34	0.04	0.36	0.06
WIMD				
Most deprived	3.79	0.26	3.27	0.20
Quintile 2	3.34	0.25	3.01	0.25
Quintile 3	2.92	0.18	2.75	0.30
Quintile 4	2.80	0.20	2.70	0.30
Least deprived	2.70	0.17	2.60	0.20

GFRs, general fertility rates; WIMD, Welsh Index of Multiple Deprivation.

The results from the ITS analysis showed that GFRs in Wales drifted from their original time series demonstrating an abrupt temporary change in GFRs during the pandemic period (a pulse change). The COVID-19 pandemic was on average associated with a decrease in GFRs of 0.22 (95% CI −0.35 to −0.10) per 4-week period between December 2020 and April 2021—showing a temporary drop in GFRs 9 months after the pandemic lasting approximately six 4-week periods. The counterfactual analysis shows that there were relative deviations in GFRs between observed and projected GFRs based on the pre-pandemic time series (figure 2). GFRs were lower than projected (using the 90% CI) in December 2020 and March and April 2021, followed by upturns in GFRs at time periods between June 2021 and December 2021 that were higher than projected, before returning to near pre-pandemic levels from the beginning of 2022 (online supplemental figure S1).

**Figure 2:**
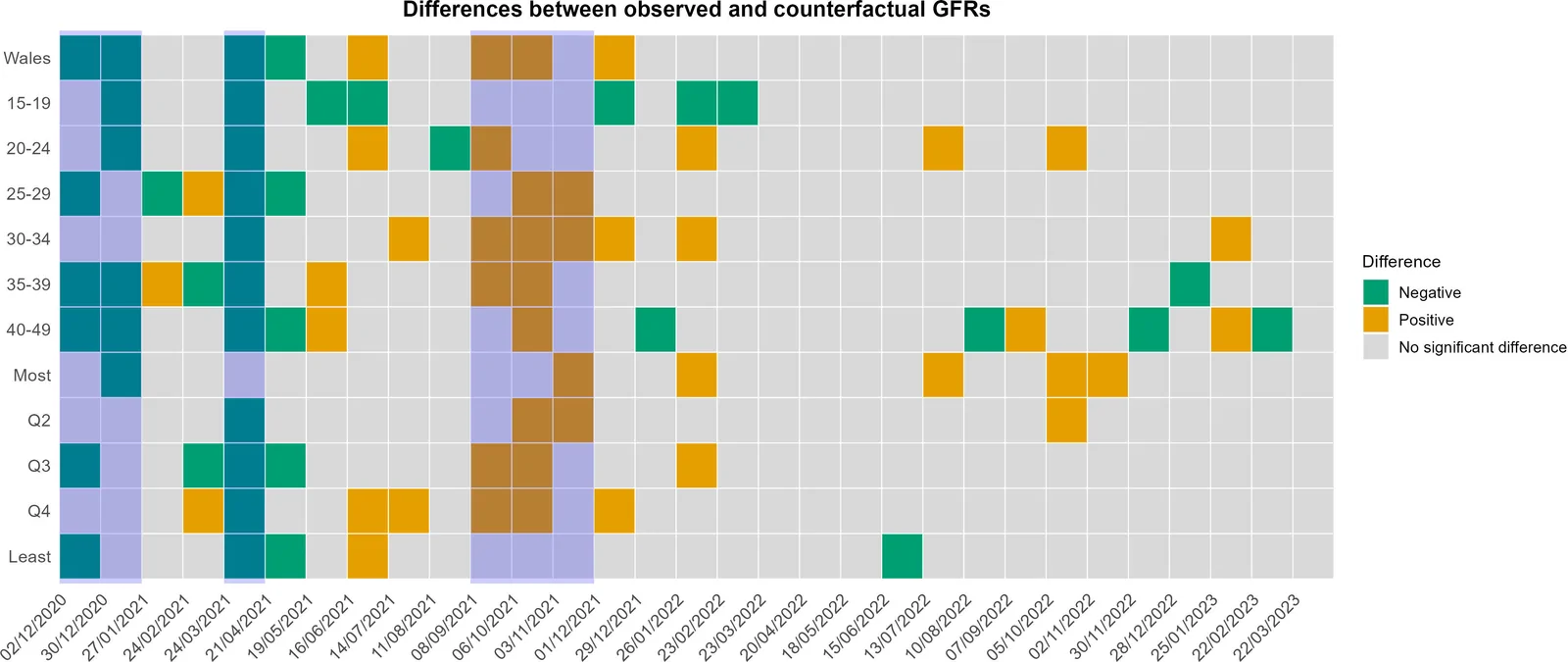
Relative difference between observed and counterfactual GFRs. Green tiles represent a significant decrease between observed and counterfactual GFRs, amber tiles represent a significant increase between observed and counterfactual GFRs and grey tiles represent no significant differences between observed and counterfactual GFRs. Blue columns represent time periods of significance. GFRs, general fertility rates.

Maternal age was associated with GFR changes during the pandemic (figure 3). Those aged 15–19 years old witnessed long-term declines in GFRs between December 2020 and March/April 2023. GFRs dropped on average by 0.16 (95% CI −0.29 to −0.03) for each 4-week period up to the end of the study period, representing an abrupt permanent level change (step change) in the time series with no bounce back to pre-pandemic levels. The counterfactual analysis also shows lower than projected GFRs for time periods between December 2020 and February 2022, with no upturns in GFRs throughout the whole time series, again suggesting a permanent decline in GFRs during the pandemic.

**Figure 3:**
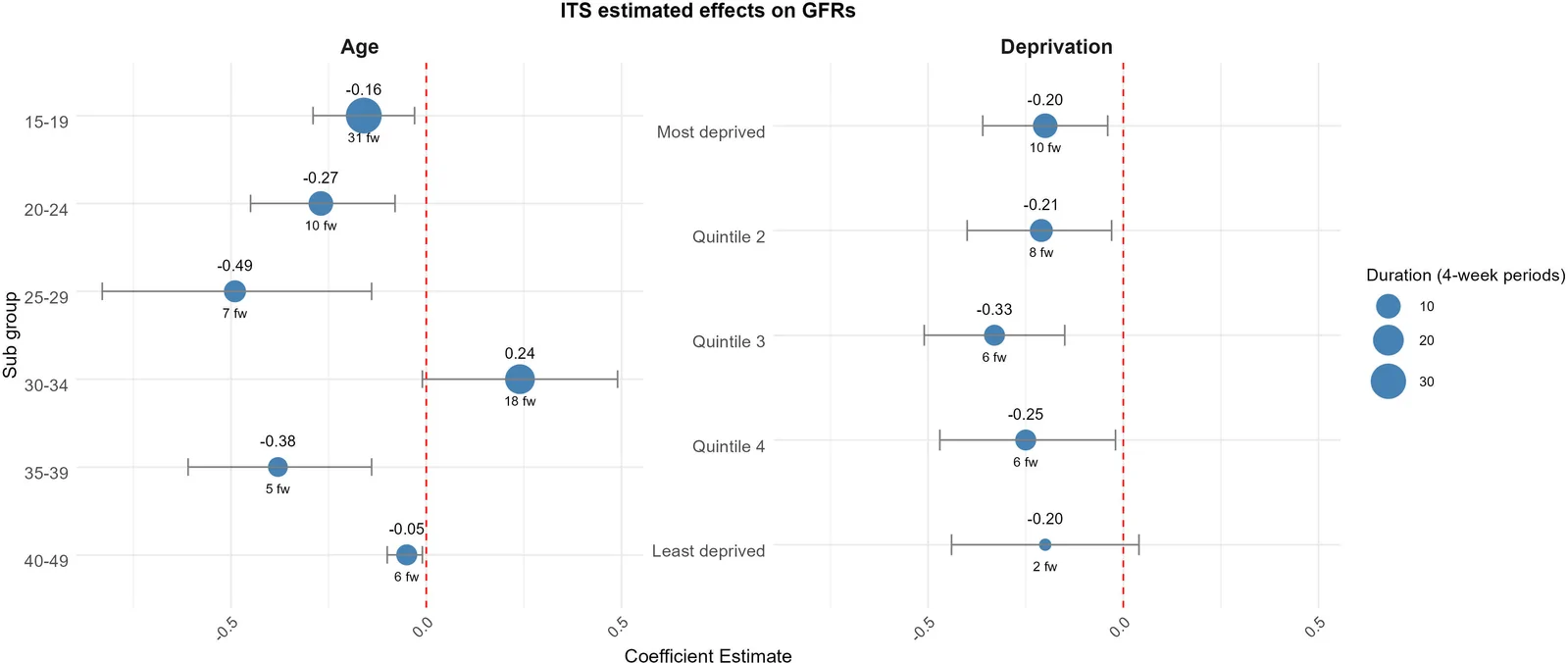
Stratified ARIMA ITS estimated coefficients with 95% CIs and impact duration. The dots represent the coefficient estimates, the error bars are the 95% CIs, and the duration is represented by the varying size in coefficient dots with key. ARIMA, Autoregressive Integrated Moving Average; GFRs, general fertility rates; ITS, interrupted time series.

Temporary impacts of the pandemic were also seen for other age categories with 20–24-year-olds, 25–29-year-olds, 35–39-year-olds and 40–49-year-olds all experiencing abrupt temporary declines to GFRs in the time series, lasting longer for those aged 20–24. On average, GFRs decreased by 0.27 (95% CI −0.45 to −0.08) per 4-week period between December 2020 and August 2021 for 20–24-year-olds, 0.49 (95% CI −0.83 to −0.14) per 4-week period between December 2020 and May 2021 for 25–29-year-olds, 0.38 (95% CI −0.61 to −0.14) per 4-week period between December 2020 and March 2021 for those aged 35–39, and 0.05 (95% CI −0.10 to −0.01) per 4-week period between December 2020 and April 2021 for 40–49-year-olds. The counterfactual analysis found that those aged 25–29, 35–39 and 40–49 experienced longer periods of lower than projected GFRs between December 2020 and April 2021, whereas those aged 25–29 only experienced lower than projected GFRs in December 2020 and March 2021. Those aged 25–29 and 35–39 experienced some upturns in GFRs at periods between May and November 2021. Yet those aged 20–24 had higher than projected GFRs at time periods lasting up to October 2022, and those aged 40–49 experienced fluctuations between lower and higher than projected GFRs up to February 2023, suggesting the pandemic had some longer impacts on fertility for these groups. There was no significant change to the time series for those aged 30–34 years suggesting the pandemic had little to no immediate impact on fertility for this age group. In the counterfactual analysis, 30–34-year-olds only experienced downturns in GFRs in March 2021 yet had longer stretches of significantly higher than projected GFRs from July 2021 to January 2022. Indicating that while localised reductions occurred for this group, the drop was not large enough or uniform across the time series to be statistically significant.

By deprivation, the pandemic was associated with temporary decreases in GFRs for most quintiles (figure 3). For the most deprived quintile, GFRs reduced on average by 0.20 (95% CI −0.36 to −0.04) per 4-week period between December 2020 and August 2021, a longer duration than any other group. Additionally, the counterfactual analysis showed that those living in the most deprived areas experienced lower than projected GFRs in December 2020 only and upturns later in 2021 and throughout 2022, later than those seen in other quintiles (figure 2). This and online supplemental figure S1 demonstrate that while there was a brief distinct drop below projected GFRs, subsequent reductions in GFRs remained within the expected range of projected variability. GFRs for quintile 2 decreased on average by 0.21 (95% CI −0.40 to −0.03) per 4-week period between December 2020 and June 2021 and decreased on average by 0.33 (95% CI −0.51 to −0.15) for quintile 3 and 0.25 (95% CI −0.47 to −0.02) for quintile 4 per 4-week period between December 2020 and April 2021, respectively. Quintile 3 experienced lower than projected GFRs between December 2020 and April 2021 and higher than projected GFRs in September and October 2021. However, quintiles 2 and 4 only experienced lower than projected GFRs in March 2021 with quintile 2 having higher than projected GFRs in October and November 2021 and October 2022, whereas quintile 4 experienced a larger stretch of higher than projected GFRs from June to December 2021. There was no significant immediate temporary change or step change in the time series for those living in the least deprived areas. Though, the counterfactual analysis shows lower than projected GFRs in December 2020 and March and April 2021 and an upturn in GFRs in June 2021 indicating localised changes in fertility for this group.

## Discussion

To our knowledge, this is the first study to explore the longer-term effects of the COVID-19 pandemic on fertility in the UK using linked population-level data. By drawing on the SAIL Databank, we were able to examine national fertility trends before, during and after the COVID-19 pandemic and more granular differences, by age and deprivation. Our findings show immediate negative effects 9 months after the start of the pandemic, when the government response measures were more severe. Consistent with other countries,^11,12,14^ Wales, at a population level, experienced a brief decline in fertility rates from December 2020 followed by a short-lived rebound. However, these shifts were not experienced equally across the population. Younger women (particularly those aged 15–19) and the more deprived quintiles saw more immediate and sustained reductions in fertility, suggesting that the pandemic measures had a greater impact on these groups by potentially deepening existing inequalities. These results emphasise the need to consider how mitigation measures and social and economic factors shape fertility responses during times of crisis.

Unlike most other age groups where temporary negative effects on fertility were found, those aged 15–19 experienced a permanent drop in GFRs that lasted the duration of the study period, from December 2020 to March/April 2023. This age group was also the only group that did not witness an upturn in GFRs later in 2021 or 2022 or indeed at any point during the study period, suggesting a long-term change to fertility trends brought on by the COVID-19 pandemic. This complements findings from Spain where the largest decreases in relative age-specific fertility rates (ASFRs) occurred among women <20 years of age. A decline in ASFR in December 2020 was largely driven by young mothers without a tertiary degree and highly educated mothers aged 34 and above.^22^ While that study did not include data beyond December 2020, another descriptive study found that births among those aged 25 and younger in Spain had not recuperated by 2022 with trends alluding to a continued deficit in births.^34^ This adverse effect on those in the younger age groups is perhaps not surprising. The national UK lockdown starting in March 2020 and subsequent measures led to a lack of social interaction and reduced opportunities for people to socialise,^35^ in turn reducing opportunities to conceive, whether intentional or accidental. As evidence from previous pandemics suggests slower recovery in the number of births for groups whose fertility was hardest hit,^36^ it might take longer than anticipated for fertility among 15–19-year-olds to bounce back to pre-pandemic trends.

Conversely, the pandemic did not significantly negatively impact GFRs for those aged 30–34; indeed, during 2021, localised GFRs were significantly higher than projected for this age group. As the average age for having a first child in the UK is 31 years old,^37^ women in this age group and to some extent those aged 25–29 might have been more reluctant to postpone or cancel childbearing, especially for their first child.^24^ The willingness of younger women to postpone childbearing to the future could explain the longer decline in GFRs and subsequent increase found in 2022 among those aged 20–24. Younger people were also more likely to lose their job or become furloughed during the pandemic than those in the older age groups, increasing financial uncertainty.^17^ Those aged 35–39, especially if they already had children, may have postponed or abandoned having subsequent children due to them juggling childcare, home schooling and working from home caused by school and nursery closures and reduced familial support, which increased the burden of care on women.^35,38,39^ For women aged 40–49 years, the initial decline in GFRs might be attributed to limited access to fertility treatments during the pandemic.^40^ Those aged 40–49 years were also the only group to experience drops in GFRs in late 2022 and early 2023 which could benefit from further exploration using more recent data.

A major influence on fertility intentions during the pandemic was changes to economic and financial situations.^19,35,41^ Our study shows for the first time that the COVID-19 pandemic did indeed reduce GFRs for those living in the most deprived areas, with immediate temporary reductions lasting from December 2020 to August 2021, longer than all other deprivation quintiles. Although GFRs for those in least deprived areas did drop initially below what was expected, there was no immediate structural change in the time series suggesting the pandemic had a relatively small impact on the fertility of the least deprived. A survey of people aged 18–34 showed that those who abandoned their fertility plans in the UK expected the pandemic to have an adverse impact on their future income.^3^ During the pandemic, the poorest in the UK experienced a reduction in living standards, bank balances and income.^42^ Additionally, savings increased more for high-income households than low-income households.^43^ While other deprivation quintiles and age groups, more notably those aged 30–34 years, witnessed increases in GFRs in late summer/autumn 2021, this was not the case for the most deprived quintile, who did not witness an upturn in rates until later in 2021 and 2022. The trend in autumn 2021 coincides with conceptions during new waves of the pandemic and subsequently, new lockdown measures in winter 2020, indicating either the creation of more favourable conditions for childbearing (working from home), differences in women/couples’ perception of the pandemic, and less apprehension about the future.^14,44^ However, later upturn in GFRs for the most deprived areas suggests that those less advantaged did not necessarily experience more favourable conditions or less apprehension. In fact, as the duration of the immediate pandemic effect on GFRs increased from the least to most deprived areas, financial pressures and uncertainties during the pandemic, especially at the start, might have been more pronounced for those from less advantaged backgrounds, disproportionately altering fertility plans for those residing in the most deprived areas.

Several other scenarios could lead to changes in fertility patterns during the earlier stages of the pandemic including postponement of weddings and ability for couples to newly cohabit.^35^ There were uncertainties surrounding the direct impact of SARS-CoV-2 on maternal and fetal health, on the health system and on maternity services.^45^ Alongside other routine services, access to reliable contraceptives, particularly long-acting reversible contraceptives such as the intrauterine device and contraceptive implant, and access to emergency contraception were also hindered during the pandemic.^46^ Access to safe abortion services, however, was facilitated by changes to abortion laws in the UK allowing women to have early medical abortions in the home via telemedicine.^47^ Considering the link between intimate partner violence (IPV) and fertility, increased instances of IPV during the pandemic may have also influenced fertility intentions for some.^48,49^ All these scenarios could contribute to changes in fertility trends during the pandemic, alongside changes to incidences of stillbirth and miscarriage, and instabilities to the population structure caused by changes to internal and external migration flows and an increase in mortality rates as a direct and indirect impact of SARS-CoV-2.

This study shows that changes in fertility during the pandemic were not distributed equally among the population. There was a shift in the composition of live births from December 2020 to at least March 2021, with group-specific durations beyond this time, namely sustained decreased GFR to March/April 2023 (the end of our study period) for those aged 15–19 years and to August 2021 for those living in the most deprived areas. Given younger women and those from less advantaged backgrounds have a higher risk of pregnancy complications and adverse maternal and neonatal health outcomes,^50,51^ these changes to the composition of births during the initial pandemic period could have produced a cohort of mothers and babies less likely to experience negative maternal and neonatal outcomes,^22,25^ despite the potential direct negative impacts of SARS-CoV-2 on pregnant women, the fetus and neonates.^52^ This is supported by reported improvements in maternal and neonatal outcomes during the pandemic, especially reductions in stillbirth incidence, low birth weight and prematurity.^53,54^ However, these improvements could be attributed to reduced physical and mental maternal stress, reduced travel, decreased air pollution and reductions in labour induction and caesarean section delivery at preterm gestation.^55^ They could also be attributed to pregnancy loss as younger women and women from more deprived areas are more likely to access abortion services.^56^ While miscarriages—either directly or indirectly affected by the pandemic—might also have a role, data, especially at a population level, are limited and research on this is scarce. If improvements in birth outcomes 9 months after the start of the pandemic might in some part be attributed to changes in the composition of births and the composition of children born as shown here, increases in observed GFRs for the most deprived area in 2022 might result in increased adverse outcomes during this time. This is the focus of our ongoing work. It remains to be seen whether changes to fertility are short-term, especially for those aged 15–19. For a deeper understanding of the impact the pandemic had on fertility, more recent linked individual-level data, including data on maternal and neonatal health outcomes, are required for more in-depth analysis.^57^ Additionally, due to potential differences in fertility patterns and composition of the population, future research should examine data from UK nations separately.

While the use of linked data enables many tens of thousands of live births to be analysed at a population scale and by age and deprivation, this study has some limitations. As date of birth is not available for anonymised data, the date of the Monday of the week of birth was used in lieu. To then alleviate the disproportionate number of Mondays in different months, 4-week periods rather than calendar months formed the basis of the analysis. Also, due to the nature of the linked data, the study uses WIMD quintile, an area-level deprivation measure, rather than individual measures of deprivation. Ethnicity data were also not available at the time of analysis. Given black and minority ethnic women have a higher risk of poor maternal and neonatal health outcomes,^58^ future work should explore this, taking into consideration potential ethnic bias within linked health data.^59^ This study provides a glimpse into the relationship between the COVID-19 pandemic and fertility. The stratified analyses using age and deprivation are limited and do not account for potential confounding effects of other factors, possible multidirectional relationships or intersectionality. Intersectional disadvantages could have influenced fertility more for specific women, for example, younger women residing in more deprived areas. Therefore, future research into fertility changes, both during the pandemic and otherwise, should account for this. This study also refrains from making causal interpretations of pandemic-related shifts in fertility.

## Conclusion

This study adds valuable insight into fertility changes among different subpopulations in the context of the COVID-19 pandemic. Other than for women aged 30–34 years and those living in the least deprived areas, GFRs were negatively impacted 9 months after the start of the pandemic, when the government response measures were more severe. Changes in GFRs during the pandemic were not evenly distributed among the population. Sustained negative impacts for the entire study period were found for those aged 15–19 years old and immediate temporary reductions up to August 2021 for those living in the most deprived areas. Government response measures and policies likely changed individuals’ socioeconomic situations and behaviours, thus potentially making it more difficult for all women other than those aged 30–34 years and those living in the least deprived areas to realise their fertility plans. This changed the composition of those giving birth during the pandemic. As those with the most sustained negative impact on fertility also have higher risks of adverse maternal and neonatal outcomes, selective fertility might have produced a healthier cohort of mothers and babies less likely to experience poor maternal and neonatal health outcomes.

## Supplementary material

10.1136/bmjph-2025-004632 online supplemental file 1

## Data Availability

Data may be obtained from a third party and are not publicly available.
