# Supplementary material for: Fertility trends and selective fertility in Wales during the COVID-19 pandemic: a population-level time series analysis
Source: BMJ Public Health. 2026 Nov 6;4(4):e004632. doi: 10.1136/bmjph-2025-004632 (PMC13756411; doi:10.1136/bmjph-2025-004632)
Supplement: Supplementary file 1 [file bmjph-4-4-s001.docx]

**Supplementary materials**

**Table S1.** ARIMA ITS analysis model specification and results.

| **Group** | **ARIMA model** | **Estimate** | **95% CI** | **AIC** | **Ljungbox test** |
| --- | --- | --- | --- | --- | --- |
| Wales | (0,1,1) (0,1,1)₁₃ | -0.22 | (-0.35, -0.10) | -112.87 | 0.579 |
| **Age** |  |  |  |  |  |
| 15-19 | (0,1,1) (0,1,1)₁₃ | -0.16 | (-0.29, -0.03) | -112.10 | 0.993 |
| 20-24 | (0,1,1) (0,1,1)₁₃ | -0.27 | (-0.45, -0.08) | 6.62 | 0.611 |
| 25-29 | (0,1,1) (0,1,1)₁₃ | -0.49 | (-0.83, -0.14) | 90.56 | 0.871 |
| 30-34 | (0,1,2) (0,1,1)₁₃ | 0.24 | (-0.01, 0.49) | 78.78 | 0.516 |
| 35-39 | (0,1,1) (0,1,3)₁₃ | -0.38 | (-0.61, -0.14) | 16.84 | 0.892 |
| 40-49 | (0,1,1) (0,1,1)₁₃ | -0.05 | (-0.10, -0.01) | -256.32 | 0.215 |
| **WIMD** |  |  |  |  |  |
| Most deprived | (0,1,2) (0,1,1)₁₃ | -0.20 | (-0.36, -0.04) | -19.06 | 0.841 |
| Quintile 2 | (0,1,1) (0,1,1)₁₃ | -0.21 | (-0.40, -0.03) | -7.48 | 0.903 |
| Quintile 3 | (2,1,1) (2,1,0)₁₃ | -0.33 | (-0.51, -0.15) | -24.47 | 0.113 |
| Quintile 4 | (0,1,1) (0,1,1)₁₃ | -0.25 | (-0.47, -0.02) | 10.74 | 0.226 |
| Least deprived | (0,1,1) (0,1,1)₁₃ | -0.20 | (-0.44, 0.04) | -37.33 | 0.711 |

**Note:** Model coefficients for pulse effects represent those with the lowest AIC.

**Figure S1.** Observed GFRs, fitted ITS ARIMA models and projected GFRs with 90% confidence intervals for Wales, deprivation quintile and age. Black line and dots are observed GFRs, blue lines are the ITS ATIMA fitted values, and orange lines are the counterfactual GFRs. The red dashed line represents the start of the COVID-19 pandemic interruption period.
